# Factors influencing health behavior practice in patients with coronary artery diseases

**DOI:** 10.1186/s12955-020-01635-2

**Published:** 2021-01-06

**Authors:** Ho Gi Jung, Ya Ki Yang

**Affiliations:** 1 Non-Governmental Organizations, Chonnam National University, Gwangju, South Korea; 2 Department of Nursing, College of Medicine, Wonkwang University, #460 Iksan-daero, Iksan city, Jeollabukdo 54538 South Korea

**Keywords:** Knowledge, Need, Health behavior, Patients, Coronary artery disease

## Abstract

**Background:**

The purpose of this study was to investigate the relationships among cardiac rehabilitation knowledge, educational need and health behavior practice in patients with coronary artery disease and explain factors influencing health behavior practice.

**Method:**

The research participants were 189 patients with coronary artery disease from general hospital located in Korea. Self-evaluation questionnaires were used to collect the data. Data was collected from January to May, 2020. Data were analyzed using descriptive statistics, independent t-test, one-way ANOVA, Pearson correlation coefficients and multiple regression with the SPSS 24.0 program.

**Results:**

There were significant positive relationships between cardiac rehabilitation knowledge and health behavior practice (*r* = .37, *p* < .001), and significant positive relationships between educational need and health behavior practice (*r* = .17, *p =* .022). Factors influencing health behavior practice were identified, the most critical predictive factor was age (≥80) (β = .52), followed by cardiac rehabilitation knowledge (β = .42), regular exercise (No) (β = −.25), family history (No) (β = .24), age (60-69) (β = .22), cohabitation (No) (β = −.20) and educational needs (β = .17). The explanation power of this model was 50% and it was statistically significant (F = 13.42, *p* < .001).

**Conclusion:**

This study suggests that cardiac rehabilitation knowledge and educational need should be considered in enhancing cardiac rehabilitation programs designed for patients with coronary artery disease.

## Background

Among the 10 leading causes of death in the world, coronary artery disease (CAD) is at the top of the chart according to the World Health Organization (WHO) [1]. In Korea, CAD is the number one cause of death as a single disease in which the mortality rate per 100,000 population was 43.4 in 2008, 60.2 in 2017, and 62.4 in 2018, thus continuously increasing. The mortality rate of CAD among heart diseases is 45.4% [2]. The purpose of CAD treatment is to stop the progression of myocardial ischemia or myocardial infarction, which typically involved drug therapy and coronary artery bypass surgery in the past. In recent years, percutaneous coronary intervention is commonly performed where a balloon or stent is used to widen a narrow or blocked passage [3]. Since the length of hospital stay is generally less than 7 days, the intervention is applied to 95% or more of patients with acute coronary syndrome [4]. According to a five-year follow-up cohort study conducted among 13,000 patients with acute coronary syndrome from 2011 to 2015, the success rate of percutaneous coronary intervention is 99.4% [5]. Despite the high success rate of the treatment, the mortality rate increased over time as the in-hospital mortality rate was 3.9% for 30 days, 4.3% for 1 year, and 8.6% for 2 years [5]. Previous studies conducted in other countries also reported that one out of 10 patients who had received percutaneous coronary intervention is rehospitalized within 30 days and has a high risk of death within 1 year [6]. Therefore, it is important to prevent the occurrence of CAD and provide early treatment and to continuously manage the disease in order to prevent the recurrence after percutaneous coronary intervention.

The major aspects of cardiac rehabilitation for the patients with CAD are drug administration, diet, exercise, smoking cessation, and stress management, for which the existing health behaviors of the subjects in their everyday life need to change [7]. Practicing health behaviors helps maintaining an ideal weight by lowering blood lipid levels and obesity index while reducing the death rate as well as negative emotional problems such as anxiety and depression [8]. Therefore, practicing health behaviors, such as regular and continued drug therapy, low-fat, low sodium, and low-cholesterol diet, regular exercise, smoking cessation, follow-up examination, and compliance with treatment plan, is a major factor for preventing the recurrence after percutaneous coronary intervention [9]. Despite consistently practicing health behaviors is recommended to prevent the recurrence and deterioration after acute treatment in patients with CAD and related studies have been conducted based on various grounds, the degree of practicing health behaviors has been reported to be low across the globe and cardiac rehabilitation programs have been proven to be ineffective in helping patients practice health behaviors, improving physical functions, and reducing the mortality rate [10]. Changing the behaviors in everyday life requires a strong determination to change the lifestyle patterns that have been performed habitually in addition to a long-term management over an extended period of time, thus being difficult to practice [11]. As realization grows of the influence of health behavior practice on recovery from CAD, most current cardiac rehabilitation programs emphasize education about the disease and treatment as a way to improve treatment adherence and health behavior practice [12]. Therefore, the factors influencing the health behavior practice need to be identified first for developing and applying an efficient cardiac rehabilitation program.

An individual’s knowledge and attitude toward such behaviors are very critical factors for understanding the behavior of humans and inducing behavioral changes [13]. In particular, knowledge related to health may change an individual’s belief that induces health-related behaviors and a change in attitude for practicing health behaviors, thereby ultimately increasing the degree of practicing health behaviors [14]. Cardiac rehabilitation knowledge refers to the knowledge related to CAD including risk factor management, physical activity, and social and psychological integrity recovery [15]. A cardiac rehabilitation educational program is initiated after the cardiac event and encompasses the individual’s interests, habits, and socioeconomic lifestyle. Active components of the program include assessment of the individual’s interest and capacity to change. Another facet of assessment is determining the individual’s learning needs. Information from a questionnaire that assesses perceived learning needs of patients can provide the basis for development of an individualized cardiac rehabilitation educational program. As the health behavior practice is strengthened as the level of knowledge is higher [16], an educational program for improving cardiac rehabilitation knowledge in patients with CAD is required in addition to supportive nursing intervention for consistently maintaining health behaviors.

With respect to health behavior practice in patients with CAD, a positive effect of providing programs on disease-related knowledge and compliance with patient role was proven in a study on disease-related knowledge and health behavior practice according to the risk factor of atherosclerosis in patients with myocardial infarction [17], and a study on the effect of cardiac rehabilitation programs provided to patients with myocardial infarction on health behavior practice [16]. Furthermore, in studies on educational demands of patients with CAD, there was a high educational demand on first aid [18] and disease-related risk factors such as diet, drug administration, everyday life, exercise, and the nature of disease [15, 19]. However, the means or methods for educating patients that can be used by nurses are limited in clinical settings; the educational content preferred by family and healthcare providers vary from that preferred by patients, and therefore, the participation in programs and educational effects are not assured [20]. Therefore, for encouraging the health behavior practice in patients with CAD, it is important to examine the level of knowledge and educational demand in terms of the disease and recovery process from the perspective of patients who are the subject of disease management [20].

In this study, cardiac rehabilitation knowledge, educational demand, and health behavior practice of patients with CAD were examined and the factors affecting the health behavior practice were identified in order to build basic materials for developing a cardiac rehabilitation program based on the demands of the subjects.

The purpose of this study is to investigate cardiac rehabilitation knowledge, educational demand, and health behavior practice of patients with CAD. The detailed purposes of this study are as follows.

- Identify cardiac rehabilitation knowledge, educational demand, and health behavior practice of patients with CAD
- Identify the difference in health behavior practice according to the general characteristics of patients with CAD
- Identify the correlation between cardiac rehabilitation knowledge, educational demand, and health behavior practice of patients with CAD
- Identify the factors affecting the health behavior practice in patients with CAD

## Methods

### Research design

This research is a descriptive study for investigating cardiac rehabilitation knowledge, educational demand, and health behavior practice of patients with CAD. And identifying the factors affecting the health behavior practice.

### Subjects

The subjects of this study were those who were admitted to the cardiovascular ward after undergoing percutaneous coronary intervention (PCI) who met the selection criteria.

The criteria for selecting research subjects are as follows.

1. Those who have undergone percutaneous coronary intervention (PCI) for coronary artery disease (myocardial infarction, angina).
2. Those who have no complications from the procedure, such as severe arrhythmia or heart failure.
3. A person who has clear consciousness and has the ability to decipher Korean and can respond to this study.
4. A person who understands the purpose of the study, accepts the participation in this study, and gives written consent.
5. Those who do not have chronic obstructive pulmonary disease, musculoskeletal disease, or disability that may affect their activities.

The subjects who are hospitalized in two university hospitals in city G and one general hospital in city I were recruited through convenience sampling. Using G*Power 3.1.4, the minimum sample size for a multiple regression analysis was calculated to be 172, considering the significance level ⍺ of 0.05, statistical power of 95%, medium effect size of 0.15, and 10 independent variables. Considering a 10% withdrawal rate, a total of 200 surveys were distributed and 195 surveys were collected. A total of 189 surveys were analyzed excluding six surveys with incomplete responses.

### Research tools

#### 1) cardiac rehabilitation knowledge

Cardiac rehabilitation knowledge refers to the knowledge related to CAD including risk factor management, physical activity, and social and psychological integrity recovery [15]. The tool for measuring cardiac rehabilitation knowledge developed by Kim and Park [17] was used in this study. The tool consists of 33 items in five subareas of nature of disease, risk factors, diet, drug administration, and exercise and everyday life. Incorrect responses and ‘Do not know’ were given 0 point and correct responses were given 1 point. The total score ranged from 0 to 33 in which the higher score indicates the higher level of cardiac rehabilitation knowledge. The reliability of this tool had Cronbach’s alpha of 0.75 at the time of development, and the Kuder-Richardson Formula 20 (KR-20) was 0.77.

#### 2) educational demand

Educational demand refers to the extent that patients with CAD wish to receive education from nurses [15]. The tool for measuring educational demand CAD developed by Jeon and Park [15] was used in this study. The tool consists of 30 items in six subareas of nature of disease, risk factors, diet, drug administration, exercise and everyday life, and follow-up management. Each item was scored based on a 5-point Likert scale in which the higher score indicated the higher educational demand. The reliability of this tool had Cronbach’s alpha of 0.87 at the time of development and 0.98 in this study.

#### 3) health behavior practice

Health behavior practice means modifying habits harmful to health while accepting and maintaining lifestyle habits that are beneficial to health [21]. The tool for measuring health behavior practice developed by Han [19] was used in this study. The tool consists of 30 items in four subareas of exercise, drug administration, diet, and follow-up management. Each item was scored based on a 5-point Likert scale in which the higher score indicated the higher level of health behavior practice. The reliability of this tool had Cronbach’s alpha of 0.86 at the time of development and 0.88 in this study.

#### 4. Data collection and ethical consideration

The data were collected from January 10 to May 31, 2020. Before collecting the data, the researchers obtained an approval from the IRB (No. 01908-SB-067) of the respective institution; a self-reporting questionnaire was used. The subjects were informed of the purpose and procedure of the research and then instructed to sign a written consent form prior to data collection for protecting the rights of the participants. The consent form was printed separately to be signed in order to allow the subjects to honestly answer the questionnaire, and then sealed in the collection envelope to be submitted. All the data were analyzed anonymously.

#### 5. Data analysis

The collected data were analyzed using SPSS 24.0, and the detailed data analysis methods are as follows.

- The subjects’ general characteristics, cardiac rehabilitation knowledge, educational demand, and health behavior practice were analyzed based on descriptive statistics.
- The difference in health behavior practice according to the general characteristics of the subjects was analyzed with the independent t-test and one-way ANOVA.
- The correlation between cardiac rehabilitation knowledge, educational demand, and health behavior practice was analyzed with the Pearson’s correlation coefficient.
- The factors influencing the health behavior practice were analyzed with multiple regression analysis.

## Results

### 1. The general characteristics of the subjects

69.3% of the subjects were male, and 30.7% were female; most of the subjects (31.7%) were in their 60s. Most of the subjects had a high school degree or higher (70.9%), and 72% of the subjects were living with a cohabitant. 78.8% of the subjects did not smoke, and most of the subjects (50.8%) had the duration of illness of less than a year. 65.6% of the subjects did not have a family history, and most of the subjects (39.7%) had a monthly income of 2 million won or higher; only 55.6% of the subjects were exercising regularly. 55.6% of the subjects had experience of receiving cardiac rehabilitation education, and most of the subjects (40.2%) had a body mass index (BMI) of less than 23. 82% of the subjects had lower-density lipoprotein (LDL) cholesterol level of 130 mg/dL or below (Table 1).

**Table 1:** Health Behavior Practice to the General Characteristics of Participants (*n* = 189)

Characteristics	Categories	N (%)	Mean (SD)	t/F	*p*	Scheffé
Sex	Male	131 (69.3)	3.09 (0.42)	−3.13	.002	
	Female	58 (30.7)	3.32 (0.47)			
Age (year)	≤49^a^	23 (12.2)	2.74 (0.61	9.90	<.001	a < c,d,e b < e
	50-59^b^	35 (18.5)	3.08 (0.39			
	60-69^c^	60 (31.7)	3.18 (0.31)			
	70-79^d^	26 (13.8)	3.26 (0.36)			
	≥80^e^	45 (23.8)	3.38 (0.48)			
Education level	≤Middle school	55 (29.1)	3.23 (0.48)	1.34	.183	
	≥High school	134 (70.9)	3.13 (0.43)			
Cohabitation	Yes	136 (72.0)	3.25 (0.39)	4.24	<.001	
	No	53 (28.0)	2.95 (0.51)			
Smoking	Yes	40 (21.2)	2.85 (0.51)	−5.26	<.001	
	No	14 (78.8)	3.25 (0.39)			
Morbid period (year)	<1	96 (50.8)	3.14 (0.49)	0.33	.718	
	1-2	30 (15.9)	3.15 (0.39)			
	≥3	63 (33.3)	3.20 (0.42)			
Family history	Yes	65 (34.4)	3.06 (0.47)	−2.31	.022	
	No	124 (65.6)	3.22 (0.43)			
Monthly income (thousand won)	<500	49 (25.9)	3.03 (0.55)	2.83	.062	
	≥500, <2000	65 (34.4)	3.20 (0.29)			
	≥2000	75 (39.7)	3.22 (0.47)			
Regular exercise	Yes	73 (38.6)	3.33 (0.32)	4.34	<.001	
	No	116 (61.4)	3.06 (0.48)			
Education experience	Yes	105 (55.6)	3.25 (0.38)	2.95	.004	
	No	84 (44.4)	3.06 (0.50)			
Body mass index (kg/m^2^)	<23^a^	76 (40.2)	3.27 (0.41)	7.87	.001	a,b>c
	≥23, <25^b^	33 (17.5)	3.26 (0.43)			
	≥25^c^	80 (42.3)	3.02 (0.46)			
LDL Chol (mg/dL)	≤130	155 (82.0)	3.22 (0.35)	2.61	.013	
	> 130	34 (18.0)	2.90 (0.70)			

*SD* Standard deviation

### 2. Cardiac rehabilitation knowledge, educational demand, and health behavior practice of the subjects

The average score of cardiac rehabilitation knowledge was 23.89 (4.59) points (out of 33), and the score was the highest in the order of risk factors, exercise and everyday life, diet, nature of disease, and drug administration. The average score of educational demand was 3.64 (0.82) points (out of 5), and the score was the highest in the order of follow-up management after diagnosis, nature of disease, drug administration, risk factors, exercise and everyday life, and diet. The average score of health behavior practice was 4.23 (0.45) points (out of 5), and the score was the highest in the order of drug administration, follow-up management, diet, and exercise (Table 2).

**Table 2:** Degrees of Variables (*n* = 189)

Variables	Mean (SD)	Min ∼ Max	Range
Knowledge of Cardiac Rehabilitation	23.89 (4.59)	10.00 ∼ 31.00	0 ∼ 38
Educational Needs	3.64 (0.82)	1.00 ∼ 5.00	1 ∼ 5
Health Behavior Practice	4.23 (0.45)	1.77 ∼ 4.23	1 ∼ 5

*SD* Standard deviation

### 3. Health behavior practice according to the general characteristics of the subjects

When the difference in health behavior practice according to the general characteristics of the subjects was analyzed, gender (t = − 3.13, *p =* .002), age (F = 9.90, *p* < .001), living with a cohabitant (t = 4.24, *p* < .001), smoking (t = − 5.26, *p* < .001), family history (t = − 2.31, *p =* .022), regular exercise (t = 4.34, *p* < .001), experience of cardiac rehabilitation education (t = 2.95, *p =* .004), body mass index (F = 7.87, *p =* .001), and LDL cholesterol (t = 2.61, *p =* .013) had a statistically significant difference (Table 1).

### 4. The correlation between cardiac rehabilitation knowledge, educational demand, and health behavior practice of the subjects

When the correlation between cardiac rehabilitation knowledge, educational demand, and health behavior practice of the subjects was analyzed, cardiac rehabilitation knowledge had a significantly positive correlation with health behavior practice (*r* = 0.37, *p* < .001), and educational demand also had a significantly positive correlation with health behavior practice (*r* = 0.17, *p =* .022). Therefore, a higher level of cardiac rehabilitation knowledge and educational demand led to a higher level of health behavior practice (Table 3).

**Table 3:** Correlations of Variables (*n* = 189)

Variables	Knowledge of Cardiac Rehabilitation	Educational Needs	Compliance of Health Behavior
	r (*p*)	r (*p*)	r (*p*)
Knowledge of Cardiac Rehabilitation	1		
Educational Needs	.08 (.278)	1	
Health Behavior Practice	.37 (<.001)	.17(.022)	1

### 5. The factors influencing health behavior practice in the subjects

In order to identify the factors influencing health behavior practice in the subjects, gender, age, whether living with a cohabitant, smoking, family history, regular exercise, experience of receiving cardiac rehabilitation education, BMI, and LDL cholesterol level among the general characteristics of the subjects that had a statistically significant correlation with cardiac rehabilitation knowledge and educational demand were input in a regression analysis formula to perform a stepwise multiple regression analysis. Gender, age, whether living with a cohabitant, smoking, family history, regular exercise, experience of receiving cardiac rehabilitation education, BMI, and LDL cholesterol level, which are nominal scales, were converted to dummy variables. Stepwise selection was applied for 189 subjects as no value was greater than the absolute value of 3 during case diagnosis. When the hypothesis of the independent variables in the regression analysis was test, the Durbin-Watson statistic had a value of 1.98, which is close 2, thus indicating that no autocorrelation was detected. The tolerance was 0.73 - 0.96, which is greater than 0.1, and the variance inflation factor (VIF) was 1.39 - 4.58, which did not exceed 10, thus indicating there is no issue with multicollinearity. Moreover, the residual distribution was even with respect to [0] when the residual of the scatter plot was analyzed, thus satisfying the assumptions of linearity and homoscedasticity. The standardized residual P-P plot showed that the residual is close to a 45^o^ line, thus satisfying the normality assumption. When the effect of independent variables on self-care behavior was analyzed, the most critical predictive factor was age (80 or above) (β = 0.52, *p* < .001), followed by cardiac rehabilitation knowledge (β = 0.42, *p* < .001), regular exercise (β = − 0.25, *p* < .001), and family history (β = 0.24, *p* < .001). The coefficient of determination (Adj R^2^) which represents the explanatory power of this model was .50, thus having 50% explanatory power, implying that the regression model is statistically significant (F = 13.42, *p* < .001) (Table 4).

**Table 4:** Factors Influencing Health Behavior Practice (*n* = 189)

Variable	B	SE	β	t	*p*
(Constant)	1.69	0.21		8.11	<.001
Knowledge of Cardiac Rehabilitation	0.04	0.01	.42	6.84	<.001
Educational Needs	0.92	0.03	.17	2.74	.007
Sex (female)^a^	0.07	0.07	.07	0.98	.331
Age (year) (50-59)^b^	−0.09	0.10	−.07	−0.87	.386
Age (year) (60-69)^b^	0.21	0.10	.22	2.17	.032
Age (year) (70-79)^b^	0.11	0.11	.08	0.99	.323
Age (year) (≥80)^b^	0.54	0.12	.52	4.66	<.001
Cohabitation (No)^c^	−0.20	0.07	−.20	−3.10	.002
Family history (No)^d^	0.22	0.06	.24	3.85	<.001
Regular exercise (No)^e^	−0.23	0.06	−.25	−3.72	<.001
F = 13.42, R^2^ = .54, Adj R^2^ = .50, *p* < .001					

Including variables in stepwise method: Knowledge of Cardiac Rehabilitation, Educational Needs, Sex^a^, Age^b^, Cohabitation^c^, Family history^d^, Regular exercise^e^ = dummy variable (reference group is male, ≤49, Yes, Yes, Yes), *SE* Standard error, *Adj* Adjusted

## Discussion

This study was conducted to examine cardiac rehabilitation knowledge, educational demand, and health behavior practice of patients with CAD and provide basic materials for developing a cardiac rehabilitation program based on the demands of the subjects. The following implications can be derived from the research results.

The average age of the subjects was 66.2 years and 69.3% of the subjects were male. These results are similar to a previous multi-institutional study conducted on acute myocardial infarction from 2011 to 2015 which reported that the average age was 64.1 years and the proportion of males was 73.5% [5]. The number of elderly patients with CAD is constantly increasing in recent years as the elderly population increases; while the success rate of treatment for the elderly is the same as that for younger adults, but the recurrence rate of the elderly is higher [22]. The elderly patients have various characteristics related to aging such as deteriorating in cognitive and physical functions, complicated diseases, and reduction in social supportive systems, which can affect their health behavior practice [22]. Furthermore, it is difficult for the elderly to change their lifestyle patterns or habits to improve their health behaviors; therefore, it is important to provide appropriate interventions by identifying the factors influencing their health behaviors in order to reduce recurrence and mortality rate. Generally males have a higher risk of heart diseases [5], but females may have a higher risk of CAD past the middle age of life [23]. Females tend to gain weight as their basal metabolic rate decreases due to changes in BMI after menopause, body fat percentage, and fat distribution as well as a reduction in estrogen level, which increases the risk of developing cardiovascular diseases. Since this study has a limitation in that only the patients hospitalized in certain regions were included as the subjects, repeated studies can be conducted by further separating the gender and age and possible to propose a study targeting outpatients. As the rate of smoking among the patients was 21.2% even after being hospitalized for CAD, interventions should be provided for the subjects who continue to smoke after being diagnosed with CAD through consultation and individualized support programs to encourage smoking cessation.

The results of this study showed that the subjects’ score of CAD knowledge was 66% which is fairly low. This finding is similar to that of a previous study which examined the knowledge of patients with CAD using the same tool [15]. In terms of the subareas, the knowledge of risk factors related to the disease was the highest, while that of drug administration was the lowest. The percentage of correct answers was 97% for ‘avoid standing for long time or lifting heavy objects’ and 94% for ‘avoid vigorous activity after a heart attack’ which are fairly high, and 21% for ‘CAD can be completely cured if drug treatment is followed properly,’ 35% for ‘sexual life can be maintained even after a heart attack,’ and 43% for ‘always carrying nitroglycerin to be taken at any time’ which are fairly low. The knowledge of the disease can provide proper guidelines and motivate the subjects having the disease to correctly practice health behaviors. Therefore, identifying the subject’s individual knowledge level and motivating the subjects to practice health behaviors based on proper knowledge of the disease can help with recovery and preventing the recurrence in patients with CAD. A cardiac rehabilitation educational program on drug administration, nature of disease, and exercise and everyday life, which had low scores of knowledge in this study, should be developed and applied so as to provide sufficient knowledge to subjects and family, followed by a study for verifying the effects of the educational program.

Recently, web-based educational programs are being developed for patients and family as a method of nursing intervention. A web-based program overcomes the drawbacks of face-to-face training which entails limitations in training time, place, one-time effect, and standardized content, while enabling the subjects to access the material at any time and individually adopt self-directed learning. Accordingly, web-based educational programs are recommended for the advantages in terms of time and cost.

The score of educational demand of the subjects was 3.64 points (out of 5), which is similar to a previous study conducted on patients with CAD [15]. In terms of the subareas, the educational demands for follow-up management after diagnosis and the nature of disease were the highest. The score of educational demand was 3.86 points for ‘the factors causing pain or failure in heart’ and 3.81 points for ‘the possibility of recurrence’ and ‘the time to revisit the hospital after being discharged’ which are fairly high, and 3.31 points for ‘cautions and point in time when they can resume sexual life,’ 3.42 points for ‘structure and function of the heart,’ and 3.43 points for ‘good and bad food for low-sodium diet’ which are fairly low. In a study conducted on health behavior experience of patients in the convalescent phase of myocardial infarction, the subjects wished to receive education that is applicable to everyday life after being discharged as well as personal consultation and education for questions they may have while practicing health behaviors, which corresponds to the findings of this study [24].

Unanswered questions may lead to obtaining information that has not been verified from unofficial support groups, thus bringing negative results. Accepting their own conditions and recognizing the limitations may be helpful in practicing health behaviors [25]. Therefore, providing the content through cardiac rehabilitation programs based on the educational demand of the subjects will be efficient in increasing the educational effect. Applying a customized educational program according to the subjects’ knowledge level and educational demand encourages the patients to actively participate in their recovery, thus ultimately accelerating the recovery and rehabilitation processes and preventing complications.

The score of the subjects’ health behavior practice is 4.23 points (out of 5), which is rather high. In terms of the subareas, health behavior practice for drug administration was the highest while that of exercise was the lowest. In terms of specific items, the score was 3.81 points for ‘did not take any medication without the doctor’s prescription’ and 3.79 points for ‘always visited the hospital on the scheduled date’ which were fairly high, and 2.46 points for ‘measured the heart rate after taking medication,’ 2.51 points for ‘measured the heart rate after performing activities,’ and 2.68 points for ‘did cool-down exercises every day’ which were fairly low. Health behavior such as measuring the heart rate can be easily performed by the subject with a simple training; therefore, pamphlets and demonstrations should be actively used for education and encouragement.

Exercising is critical for improving heart functions and risk factors of CAD, but lack of knowledge on specific types of exercise or cautions can lead to avoiding exercises due to the fear of having a heart attack. Thus, accurate information and knowledge on exercises need to be provided. There is a vast amount of information on diet and exercise available on TV or internet media such as YouTube, but patients may acquire incorrect knowledge due to the overflow of information that has not been verified as true. Therefore, accurate information should be provided through educational materials on cardiac rehabilitation composed by healthcare professionals including nurses. In recent times, a variety of applications are being developed in which the health information of the subjects are registered and reflected in real time [26, 27], which allows the health management status of the subjects such as the heart rate or drug administration to be identified and managed in real time, thus being effective as nursing intervention.

In terms of health behavior practice according to the general characteristics of the subjects, gender (t = − 3.13, *p =* .002), age (F = 9.90, *p* < .001), living with a cohabitant (t = 4.24, *p* < .001), smoking (t = − 5.26, *p* < .001), family history (t = − 2.31, *p =* .022), regular exercise (t = 4.34, *p* < .001), experience of cardiac rehabilitation education (t = 2.95, *p =* .004), body mass index (F = 7.87, *p =* .001), and LDL cholesterol (t = 2.61, *p =* .013) had a statistically significant difference. Based on a study by Burg et al. which reported that the morbidity rate of a socially isolated group is four times higher while the group with a spouse had a lower mortality rate along with a faster recovery period [28], repeated studies can be conducted in the future to examine whether having a spouse (cohabitant) results in the difference in health behavior practice in men and women. It is very crucial to exercise regularly since physical activities have been found to be a major predictive factor of rehospitalization and reduction in mortality rate as they lessen re-coarctation of a stent in patients with CAD by increasing the number of endothelial cells to promote revascularization of ischemic and damaged blood vessels [29]. If the patient who had received percutaneous coronary intervention for acute myocardial infarction is obese, the probability of re-coarctation is at least two times higher [30], but another study on the mortality rate of patients with heart diseases reported that the mortality rate of obese patients is not different from that of patients having normal weight [31], which indicates that further studies are required to examine the effect of obesity on the mortality rate of CAD.

The results of this study imply that education and training programs that are personalized and customized for age group, gender, or severity of disease need to be developed and applied by reflecting the general characteristics such as gender, age, and experience of receiving cardiac rehabilitation education as well as disease-related characteristics such as BMI and cholesterol level in order to promote health behavior practice in patients with CAD. However, repeated studies are needed to investigate health behaviors by varying the region and scale since the subjects of this study are limited to patients who are hospitalized in certain regions.

The subjects’ cardiac rehabilitation knowledge and educational demand have a statistically significant correlation with health behavior practice. It corresponds to the findings of a study which reported a higher level of health behavior practice was observed when the cardiac rehabilitation knowledge level was higher [32]. The subjects’ self-efficacy increases and thus enhances health behavior if education on disease and disease management is systematically provided through healthcare professionals such as nurses to ensure the patients have proper knowledge on their disease and disease management [17, 32].

This study showed that cardiac rehabilitation knowledge and educational demand explains health behavior practice 50% along with age, whether living with a cohabitant, family history, and regular exercise. In particular, cardiac rehabilitation knowledge and age (≥80) were the most critical factors of health behavior practice, which corresponds to a previous study which emphasized the importance of improving cardiac rehabilitation knowledge to encourage the patients with CAD to practice health behaviors [16, 17, 21]. As nursing invention for improving health behavior practice in patients with CAD, it is important to expand and promote the opportunity to learn about cardiac rehabilitation and to encourage the changes in patients to adopt healthy lifestyles and habits.

This study has a significance in that it investigated the correlation between cardiac rehabilitation knowledge, educational demand, and health behavior practice of patients with CAD, and verified the necessity of providing educational programs on cardiac rehabilitation based on the educational demand of the subjects as an intervention strategy to improve health behavior practice in patients with CAD.

By referring to the results of this study, education and consultation should be provided for the items with high educational demand and low levels of knowledge and health behavior practice in patients with CAD, while encouraging the patients to adopt proper health management in everyday life. Nurses should recognize that providing health education is an important part of their work, and thus establish patient-oriented and integrated cardiac rehabilitation educational programs based on a multidisciplinary approach and take initiatives in providing such education to patients. Meanwhile, as the degree of practicing health behavior may increase at first but decrease over time after a cardiac rehabilitation program was provided [20], a refresher course can be offered to the subjects one to 2 years after they are discharged when the degree of practice health behavior starts to decrease as a means of encouragement.

## Conclusions

This study aimed to examine the cardiac rehabilitation knowledge, educational demand, and health behavior practice of patients with CAD and to identify the factors affecting the health behavior practice in order to provide basic materials for developing a cardiac rehabilitation program for improving the health of the patients with CAD. The study confirmed that cardiac rehabilitation knowledge and educational demand of patients with CAD are major influential factors for practicing health behavior.

The following proposals are made based on the findings of this study. First, the subjects of this study were the patients with CAD from only certain regions; thus, repeated studies are proposed to take the characteristics of hospitals, such as region and size, into consideration when sampling the subjects. Second, the educational programs for the patients with CAD should include content reflecting cardiac rehabilitation knowledge, educational demand, and health behavior practice which were examined in this study. Especially, by reflecting the items with low levels of cardiac rehabilitation knowledge and health behavior practice, those with high educational demand, and the items with a statistically significant difference in the general characteristics, a cardiac rehabilitation program personalized for each patient based on their demand and that improves the knowledge of the patients with CAD should be provided.

## Data Availability

The datasets during and / or analysed during the current study available from the corresponding author on reasonable request.
